# Supplementary material for: Paternal dietary macronutrient balance and energy intake drive metabolic and behavioral differences among offspring
Source: Nat Commun. 2024 Apr 6;15:2982. doi: 10.1038/s41467-024-46782-y (PMC10998877; doi:10.1038/s41467-024-46782-y)
Supplement: Supplementary file 1 — Supplementary Information [file 41467_2024_46782_MOESM1_ESM.pdf]

Supplementary Information

Supplementary Figures

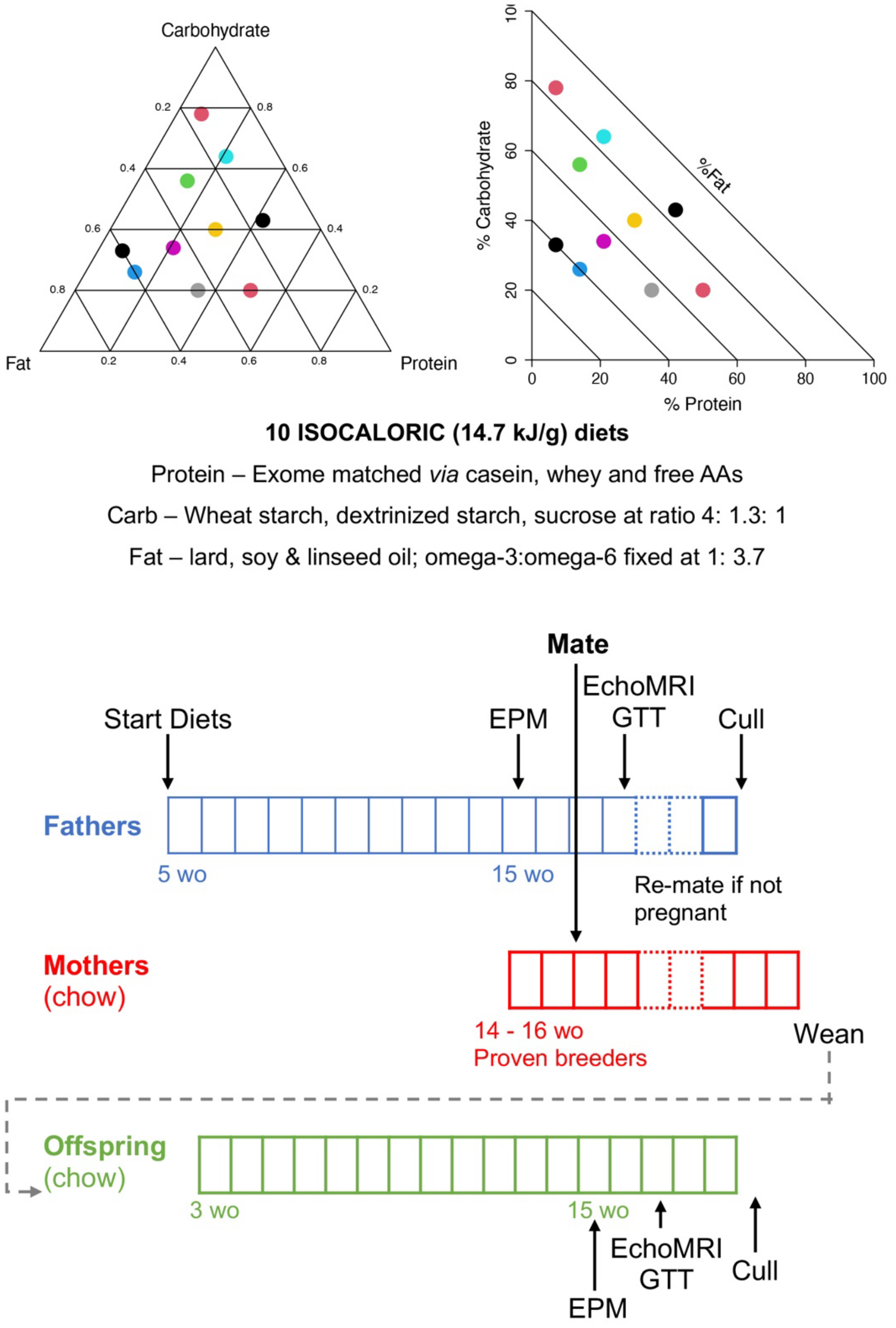

Supplementary Fig. 1. Experimental design and timeline. Ten isocaloric (14.7 kJ/g), semi-pure diets, made from the same ingredients (Table S1) with proportions varied to cover a physiologically relevant range of macronutrients, were fed to male C57BL/6J mice from 5 weeks of age. After 10 weeks on experimental diets (15 weeks of age), male behavior in an Elevated Plus Maze (EPM) was assayed. After 12 weeks on experimental diet (17 weeks of age), males were mated to age matched C57BL/6J females fed a standard chow diet. At 18 weeks of age male body composition was measured using EchoMRI, and metabolic health

measured using an oral glucose tolerance test (GTT). Mating pairs were given two more chances to reproduce if the first pairing was unsuccessful. Up to 4 male and 4 female offspring from each litter were weaned onto standard chow at 3 weeks of age. Behavioral (EPM) and metabolic (EchoMRI and GTT) assays were repeated on up to 2 male and 2 female offspring from each litter at the same age as fathers.

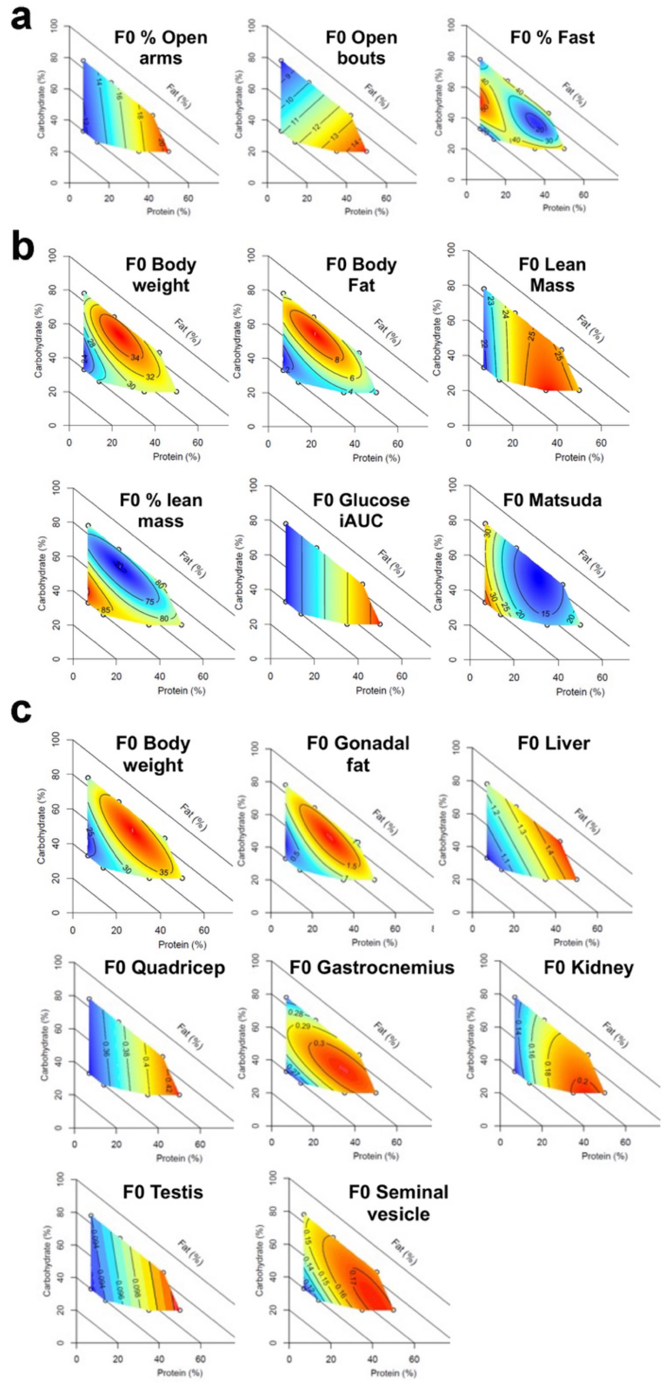

Supplementary Fig. 2. Direct effect of treatment diets on significant paternal traits not shown in the main figures: (a) behavioral traits at 15 weeks of age, n=60, (b) metabolic traits measured at 17 weeks of age, n=60, (c) organ weights at cull, n=57.

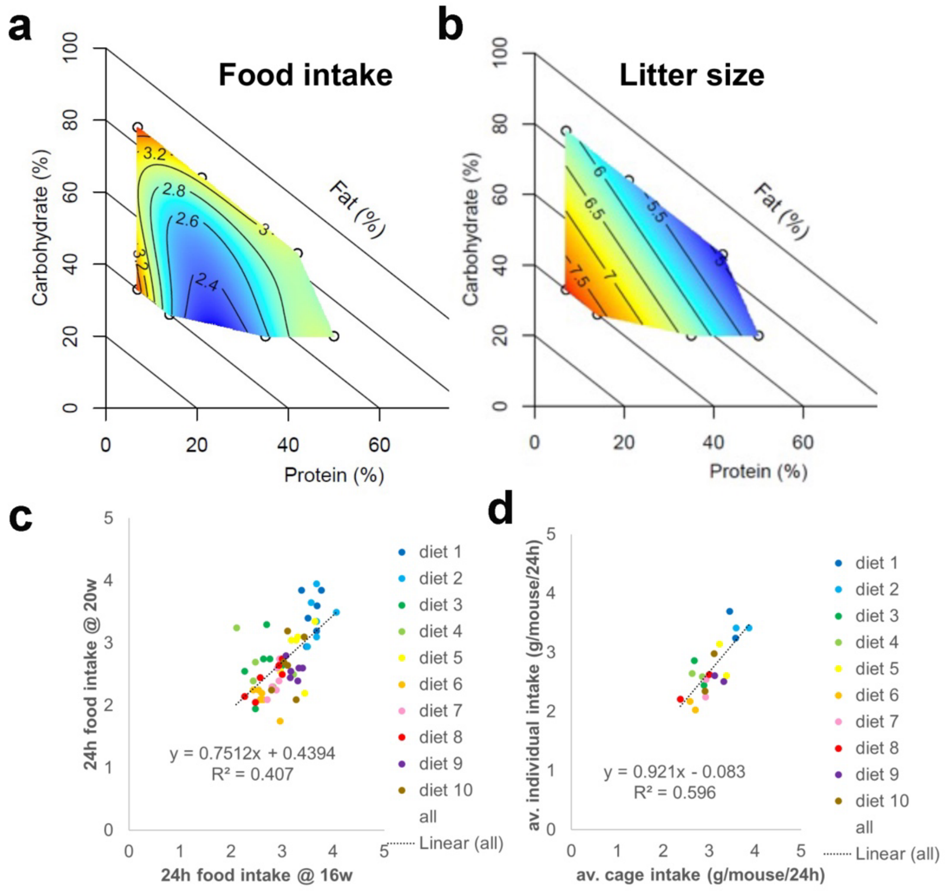

Supplementary Fig. 3. Impact of paternal diets on moderating factors used in Generalized Additive Models: (**a**) Food intake (g/day), n=60, (**b**) litter size, n=60, (**c**) relationship between the replicate individual 24h food intake measures collected at 16 and 20 weeks of age (average of both measures used in analyses), n=60, (**d**) consistency of individual food intake measures with cage-based food intake measures recorded prior to separation for mating, n=20.

Body fat – Female offspring

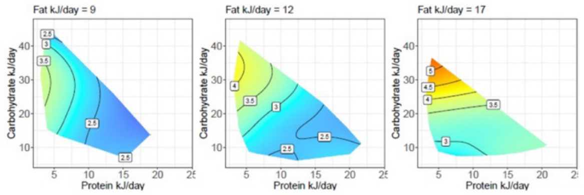

% Body fat – Female offspring

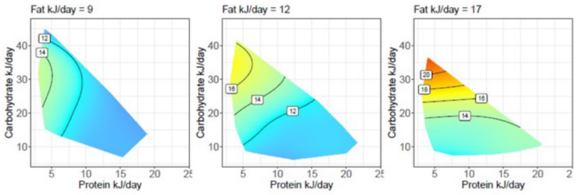

Body fat – Male offspring (not statistically significant)

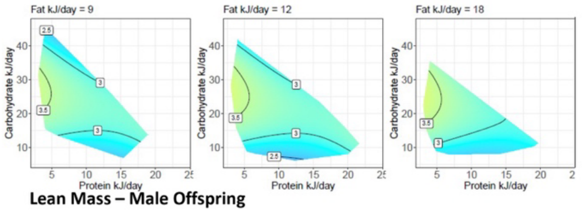

Lean Mass – Male Offspring

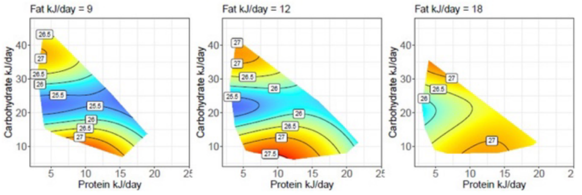

% Lean Mass – Male Offspring (not statistically significant)

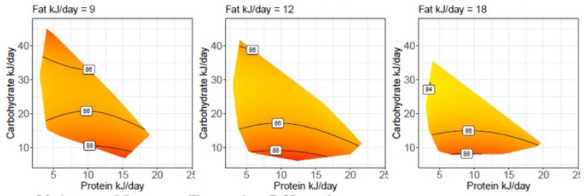

% Lean Mass – Female Offspring

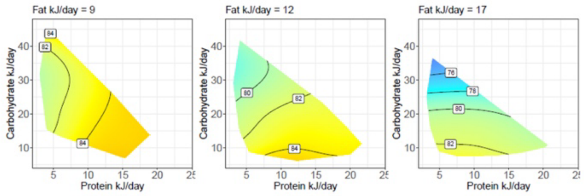

Glucose iAUC – Male Offspring

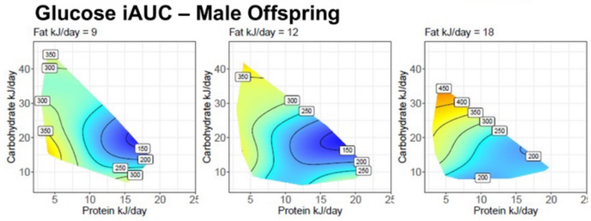

Glucose iAUC – Female Offspring (not statistically significant)

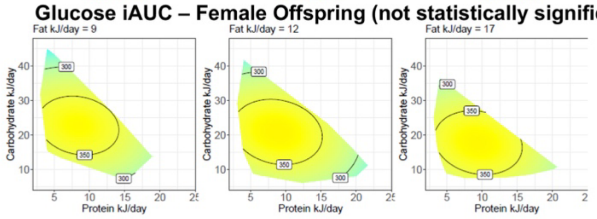

Liver triglycerides – Female Offspring

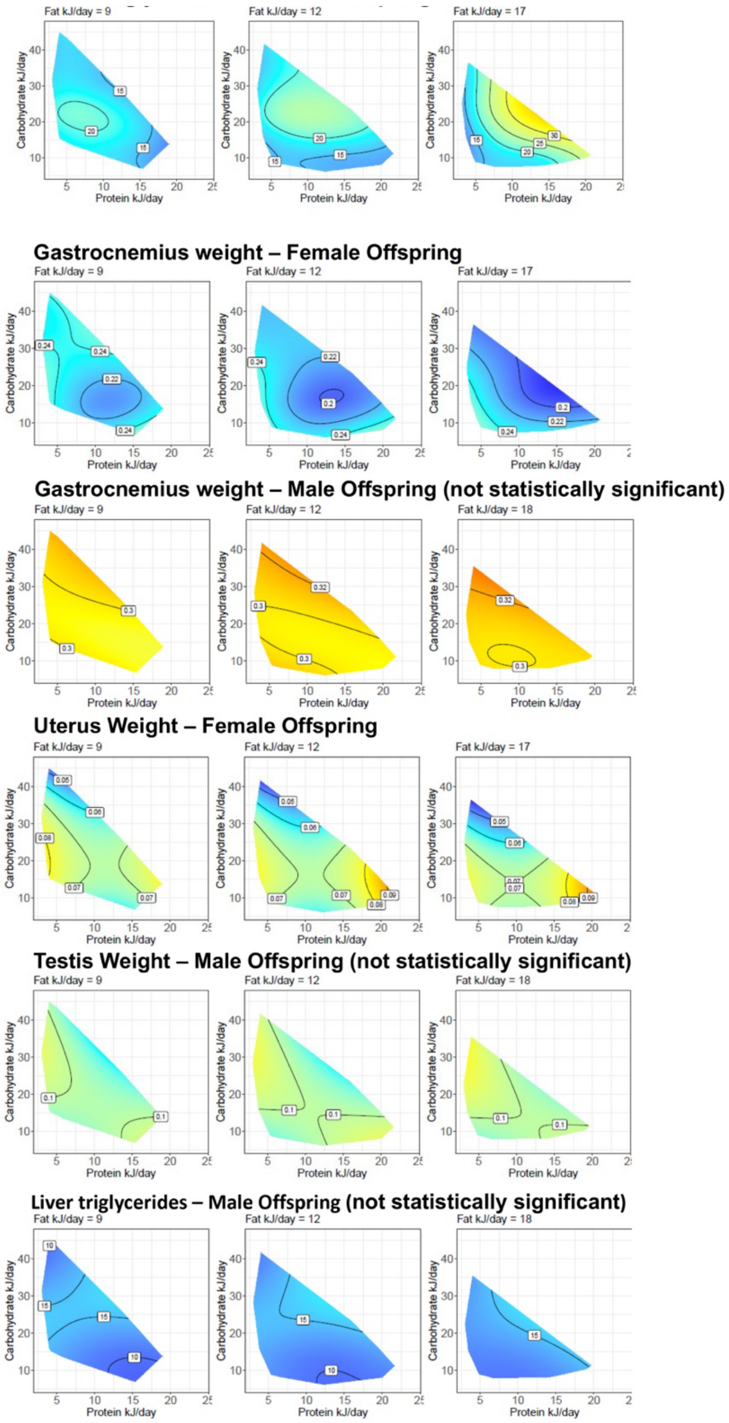

Supplementary Fig. 4. Response surfaces derived from GAM model predictions, showing offspring traits plotted against nutrient intake of fathers (x-axis: paternal protein consumption (kJ/day), y-axis: paternal carbohydrate consumption (kJ/day)). Three slices of the nutrient space are shown, cut through the Q1, Q2, and Q3 of fat intake. Color scale indicates offspring trait values (blue = minimum, red = maximum) with isolines showing the model predicted response. All traits where incorporating paternal nutrient intake revealed significant effects of paternal diet that were not evident from mixture models are shown. The response surfaces of both sexes are shown even where the response to paternal diet was only significant in one sex

for comparative purposes. Females, n=50; Females Liver Triglycerides, n=49; Males, n=51; Males Liver Triglycerides, n=50.
