# Supplementary material for: Paternal dietary macronutrient balance and energy intake drive metabolic and behavioral differences among offspring
Source: Nat Commun. 2024 Apr 6;15:2982. doi: 10.1038/s41467-024-46782-y (PMC10998877; doi:10.1038/s41467-024-46782-y)
Supplement: Supplementary file 3 — Description of Additional Supplementary Files [file 41467_2024_46782_MOESM3_ESM.pdf]

Description of Additional Supplementary Files

Supplementary Data 1. Diet ingredients

Supplementary Data 2. AIC values and mixture model summaries for all variables

Supplementary Data 3. Raw data F0

Supplementary Data 4. Raw data F1 female offspring

Supplementary Data 5. Raw data F1 male offspring

Supplementary Data 6. Chi-sq comparison of GAM models for all variables
