# Supplementary material for: Paternal dietary macronutrient balance and energy intake drive metabolic and behavioral differences among offspring
Source: Nat Commun. 2024 Apr 6;15:2982. doi: 10.1038/s41467-024-46782-y (PMC10998877; doi:10.1038/s41467-024-46782-y)
Supplement: Supplementary file 5 — Reporting Summary [file 41467_2024_46782_MOESM5_ESM.pdf]

nature portfolio

Corresponding author(s): Romain BARRES
Stephen SIMPSON

Last updated by author(s): 6 december 2023

Reporting Summary

Nature Portfolio wishes to improve the reproducibility of the work that we publish. This form provides structure for consistency and transparency in reporting. For further information on Nature Portfolio policies, see our Editorial Policies and the Editorial Policy Checklist.

Statistics

For all statistical analyses, confirm that the following items are present in the figure legend, table legend, main text, or Methods section.

n/a	Confirmed	
☐	☒	The exact sample size (*n*) for each experimental group/condition, given as a discrete number and unit of measurement
☐	☒	A statement on whether measurements were taken from distinct samples or whether the same sample was measured repeatedly
☐	☒	The statistical test(s) used AND whether they are one- or two-sided<br>*Only common tests should be described solely by name; describe more complex techniques in the Methods section.*
☐	☒	A description of all covariates tested
☐	☒	A description of any assumptions or corrections, such as tests of normality and adjustment for multiple comparisons
☐	☒	A full description of the statistical parameters including central tendency (e.g. means) or other basic estimates (e.g. regression coefficient) AND variation (e.g. standard deviation) or associated estimates of uncertainty (e.g. confidence intervals)
☐	☒	For null hypothesis testing, the test statistic (e.g. *F*, *t*, *r*) with confidence intervals, effect sizes, degrees of freedom and *P* value noted<br>*Give P values as exact values whenever suitable.*
☒	☐	For Bayesian analysis, information on the choice of priors and Markov chain Monte Carlo settings
☐	☒	For hierarchical and complex designs, identification of the appropriate level for tests and full reporting of outcomes
☐	☒	Estimates of effect sizes (e.g. Cohen's *d*, Pearson's *r*), indicating how they were calculated

Our web collection on statistics for biologists contains articles on many of the points above.

Software and code

Policy information about availability of computer code

Data collection	No software was used for data collection
Data analysis	R (v 4.2.0) using mixture models (mixexp package)

For manuscripts utilizing custom algorithms or software that are central to the research but not yet described in published literature, software must be made available to editors and reviewers. We strongly encourage code deposition in a community repository (e.g. GitHub). See the Nature Portfolio guidelines for submitting code & software for further information.

Data

Policy information about availability of data

All manuscripts must include a data availability statement. This statement should provide the following information, where applicable:

- Accession codes, unique identifiers, or web links for publicly available datasets
- A description of any restrictions on data availability
- For clinical datasets or third party data, please ensure that the statement adheres to our policy

Source data are provided with this paper.

Research involving human participants, their data, or biological material

Policy information about studies with human participants or human data. See also policy information about sex, gender (identity/presentation), and sexual orientation and race, ethnicity and racism.

Reporting on sex and gender	N/A
Reporting on race, ethnicity, or other socially relevant groupings	N/A
Population characteristics	N/A
Recruitment	N/A
Ethics oversight	N/A

Note that full information on the approval of the study protocol must also be provided in the manuscript.

Field-specific reporting

Please select the one below that is the best fit for your research. If you are not sure, read the appropriate sections before making your selection.

- [x] Life sciences
- [ ] Behavioural & social sciences
- [ ] Ecological, evolutionary & environmental sciences

For a reference copy of the document with all sections, see nature.com/documents/nr-reporting-summary-flat.pdf

Life sciences study design

All studies must disclose on these points even when the disclosure is negative.

Sample size	Sample size was determined based on previous studies reporting paternal effects under dietary stress. Sixty 4-week-old male mice were acquired, housed in groups of 3 animals per cage, and allowed to acclimate for 4 days before being switched from standard chow (Specialty Feeds standard diet SF00-100, 14.0 MJ/kg, 23% protein, 11% fat) to experimental diets fed ad libitum (n = 6/diet). After 10 weeks on diet, anxiety-like behavior was assayed in an Elevated Plus Maze (details below), and then males were separated into individual cages in preparation for mating. After 12 weeks on diet, males were mated to age-matched, fertility-proven (previously produced a litter) C57BL/6J females (details below). Unsuccessful pairs were given a second chance at mating 2 weeks later (n = 13), and a final opportunity 2 weeks later if still unsuccessful (n = 10). Body composition and glucose tolerance was assayed at 18 weeks of age (details below). Males were culled after the successful birth of pups or after the third mating attempt. Large litters were culled to 8 pups at 3 days old to minimize litter size effects, and pups were weaned on to standard chow (SF00-100) at P21. Behavior and metabolic assays were performed on offspring at the same age as they were performed on fathers for comparative purposes. Two males and 2 females from each litter were assayed where possible (n = 99 male and 94 female offspring), with the average trait values per sex per litter used in analyses. Litter mates were kept as companion animals to maintain group housing requirements as per ethics permit.
Data exclusions	No data exclusion
Replication	This work represent results from one cohort described in the sample size, there was no replication of the cohort.
Randomization	Animals were distributed by ancestor diets
Blinding	No blinding was made

Reporting for specific materials, systems and methods

We require information from authors about some types of materials, experimental systems and methods used in many studies. Here, indicate whether each material, system or method listed is relevant to your study. If you are not sure if a list item applies to your research, read the appropriate section before selecting a response.

Materials & experimental systems

n/a	Involved in the study
☒	☐ Antibodies
☒	☐ Eukaryotic cell lines
☒	☐ Palaeontology and archaeology
☐	☒ Animals and other organisms
☒	☐ Clinical data
☒	☐ Dual use research of concern
☒	☐ Plants

Methods

n/a	Involved in the study
☒	☐ ChIP-seq
☒	☐ Flow cytometry
☒	☐ MRI-based neuroimaging

Animals and other research organisms

Policy information about studies involving animals; ARRIVE guidelines recommended for reporting animal research, and Sex and Gender in Research

Laboratory animals	C57BL/6J (JAX strain code 000664)
Wild animals	No wild animals were used in this study
Reporting on sex	Results reported on both sexes
Field-collected samples	No field-collected samples
Ethics oversight	Local ethics committee of the University of Sydney animal ethics committee (project number 2019/1610)

Note that full information on the approval of the study protocol must also be provided in the manuscript.

Plants

Seed stocks	*Report on the source of all seed stocks or other plant material used. If applicable, state the seed stock centre and catalogue number. If plant specimens were collected from the field, describe the collection location, date and sampling procedures.*
Novel plant genotypes	*Describe the methods by which all novel plant genotypes were produced. This includes those generated by transgenic approaches, gene editing, chemical/radiation-based mutagenesis and hybridization. For transgenic lines, describe the transformation method, the number of independent lines analyzed and the generation upon which experiments were performed. For gene-edited lines, describe the editor used, the endogenous sequence targeted for editing, the targeting guide RNA sequence (if applicable) and how the editor was applied.*
Authentication	*Describe any authentication procedures for each seed stock used or novel genotype generated. Describe any experiments used to assess the effect of a mutation and, where applicable, how potential secondary effects (e.g. second site T-DNA insertions, mosiacism, off-target gene editing) were examined.*
